# Association between statin use and Alzheimer’s disease with dose response relationship

**DOI:** 10.1038/s41598-021-94803-3

**Published:** 2021-07-27

**Authors:** Su-Min Jeong, Dong Wook Shin, Tae Gon Yoo, Mi Hee Cho, Wooyoung Jang, Jinkook Lee, SangYun Kim

**Affiliations:** 1 Department of Family Medicine, Samsung Medical Center, Supportive Care Center, Samsung Comprehensive Cancer Hospital, 81 Irwon-Ro, Gangnam-gu, Seoul, Republic of Korea; 2 Department of Clinical Research Design & Evaluation, Samsung Advanced Institute for Health Science & Technology (SAIHST), Sungkyunkwan University, Seoul, Republic of Korea; 3 Department of Family Medicine, Hongseong Medical Center, Hongseong, Republic of Korea; 4 Samsung C&T Medical Clinic, Kangbuk Samsung Hospital, Seoul, Republic of Korea; 5 Department of Neurology, Gangneung Asan Hospital, University of Ulsan College of Medicine, Gangneung, Republic of Korea; 6 Department of Economics & Center for Economic & Social Research, Los Angeles, & RANC Corporation, University of Southern California, Santa Monica, CA USA; 7 Department of Neurology, Seoul National University Bundang Hospital, Seoul National University College of Medicine, Seongnam, Republic of Korea

**Keywords:** Epidemiology, Neurodegenerative diseases

## Abstract

This study aimed to determine the dose–response relationship between the levels of statin exposure and the incidence of Alzheimer’s disease (AD). We included 119,013 Korean adults (≥ 60 years old) using a database from the Korean National Health Insurance Service (2002–2013). Statin exposure was treated as a time-varying variable. Incidence of AD was defined by the first claim code for AD with anti-Alzheimer drugs. AD occurred in 9467 cases during a median 7.2 years of follow-up. Overall, statin use was not associated with an increased risk of AD incidence [adjusted hazard ratio (aHR) = 1.04; 95% confidence interval (CI) = 0.99–1.10]. When examined by level of statin exposure, statin prescription < 540 days during a 2-year window time was associated with a higher risk for incidence of AD compared to statin non-use. However, days of prescription ≥ 540 and cumulative defined daily dose ≥ 540 of statin were associated with decreased risk of AD [aHR (95% CI) = 0.87 (0.80–0.95) and 0.79 (0.68–0.92), respectively]. Our findings indicate that less persistent statin use is associated with increased risk of AD, whereas persistent and adherent statin use is associated with decreased risk of AD.

## Introduction

Dementia is a progressive neurodegenerative disease that not only profoundly affects the health and well-being of patients and caregivers, but is also a significant public health problem^1^. The number of people living with dementia is projected to be about 131 million by 2050, given the rapidly aging population worldwide^2^. Alzheimer’s disease (AD) is the most common type of dementia characterized by a progressive decline in cognitive function in particular the memory domain^3^.

Statins are 3-hydroxy-3-methylglutaryl coenzyme A (HMG-CoA) reductase inhibitors widely prescribed for primary and secondary prevention of cardiovascular disease^4,5^. As one of the most commonly used drugs, there has been concern that statin use has a long-term adverse effect, including cognitive dysfunction and dementia. In 2012, the US Food and Drug Administration (FDA) raised concerns about the adverse cognitive effects of statins, such as memory loss, based on case reports^6^. Concerns about adverse cognitive effects could be a reason to avoid this life-saving preventive medication and to discontinue it once prescribed. Contrary to the FDA’s warning, a systematic review of the literature^7^, including evidence from a randomized trial^8,9^, did not suggest any adverse effect of statin use on cognition, although the amount and strength of the available evidence are limited. However, there are reports that the public avoids or discontinues statin use due to fear of memory loss or dementia^10^.

A recent meta-analysis of observational studies suggested that statin use is associated with decreased risk of AD as well as vascular dementia^11^. Prospective cohort studies have shown inconsistent results: some studies showed a marked reduction in dementia incidence with statin use^12–14^, while others showed a higher risk of dementia with statin use without statistical significance^15–17^. However, previous studies on the effect of statin use on dementia outcome are limited in the following aspects: (1) Most studies classified subjects as either a statin user or non-user at baseline without considering the time-varying nature of statin consumption^12–14,16,18^. However, in the real world, the status of statin use can change considerably. Many patients discontinue statin use, and there is variability in drug adherence. Conversely, statin use dramatically increased during the last two decades^19^, and many statin non-users turned into statin users, (2) The dose–response effect was not examined mainly because statin use was determined by a questionnaire^12–16^, and the number of study participants was too small (110–582 cases among 8 prospective cohort studies)^12–17^, (3) Most of the study durations were too short (mean duration of follow-up ≤ 5 years)^12,13,15,16^, and (4) a Taiwanese study with the largest sample size suffered from lack of potential confounder information, such as baseline cholesterol level^18^. In this study, we aimed to evaluate the association of statin use with incidence of AD considering the time-varying status of statin use along with the dose–response relationship.

## Results

### Baseline characteristics

The mean age of the study population was 66.0 [standard deviation (SD), 4.9] years, and 48.1% of the subjects were male (Supplementary Table 1). In total, 7.2% of the subjects were statin users in 2002–2003. The mean (SD) cholesterol levels at baseline were 202.2 (39.5), 200.4 (38.2), and 225.1 (48.0) in all subjects, statin non-users, and statin users, respectively. Statin users (65.6%) were more likely to be women compared to statin non-users (50.8%). In addition, statin users tended to have hypertension or diabetes more often than statin non-users. Statin prescription rates increased from 7.2% in 2002–2003 to 27.5% in 2010–2011 (Table 1). The proportions of statin prescriptions ≥ 540 days and ≥ 540 cDDD among statin users during the 2-year time window also increased from 9.1% and 0.4% in 2002–2003, respectively, to 14.4% and 44.4% in 2010–2011.

**Table 1:** Distribution of statin use for each 2-year time window.

	2002–2003	2004–2005	2006–2007	2008–2009	2010–2011	2012–2013
AD incidence^a^ (n)	NA	475	930	1,835	2,794	3,433
Statin user, n (%)	8,621 (7.2)	13,404 (11.6)	19,281 (17.4)	23,645 (22.5)	26,884 (27.5)	NA
Non-user, n (%)	110,392 (92.8)	102,135 (88.4)	91,617 (82.6)	81,370 (77.5)	71,004 (72.5)	NA
**Duration of medication use, days, n (%)**						
< 90	3,725 (43.2)	4,605 (34.4)	4,962 (25.7)	4,696 (19.9)	4,225 (15.7)	NA
90–180	1,754 (20.4)	2,502 (18.7)	3,095 (16.1)	3,197 (13.5)	2,787 (10.4)	NA
180–365	1,653 (19.2)	2,759 (20.6)	3,867 (20.1)	4,431 (18.7)	4,481 (16.7)	NA
365–540	700 (8.1)	1,437 (10.7)	2,436 (12.6)	3,166 (13.4)	3,468 (12.9)	NA
≥ 540	789 (9.1)	2,101 (15.7)	4,921 (25.5)	8,155 (34.5)	11,923 (44.4)	NA
**cDDD of medication use, n (%)**						
< 90	5,557 (64.5)	6,541 (48.8)	6,817 (35.4)	6,730 (28.5)	6,139 (22.8)	NA
90–180	1,573 (18.2)	2,560 (19.1)	3,425 (17.8)	3,936 (16.7)	3,937 (14.6)	NA
180–365	1,235 (14.3)	3,096 (23.1)	5,148 (26.7)	6,723 (28.4)	9,220 (34.3)	
365–540	222 (2.6)	904 (6.7)	2,562 (13.3)	3,588 (15.2)	3,719 (13.8)	NA
≥ 540	34 (0.4)	303 (2.3)	1,329 (6.9)	2,668 (11.3)	3,869 (14.4)	NA

*AD* Alzheimer’s disease, *cDDD* cumulative defined daily dose, *NA* non-applicable.

^a^Total AD cases = 9467.

### Serum cholesterol levels and incidence of Alzheimer’s disease risk

Compared to moderate total cholesterol levels (200–240 mg/dL), low cholesterol levels (< 160 mg/dL) and high cholesterol levels (≥ 300 mg/dL) were associated with an increased risk of AD with a U-shaped curve (Supplementary Table 2) (adjusted hazard ratio [aHR] = 1.12, 95% confidence interval [CI] = 1.05–1.20 in < 160 mg/dL and aHR = 1.22, 95% CI = 1.05–1.42 in ≥ 300 mg/dL). This U-shaped association was more prominent among statin non-users at baseline (2002–2003).

### Association of statin use and dose with incidence of AD

**Figure 1:**
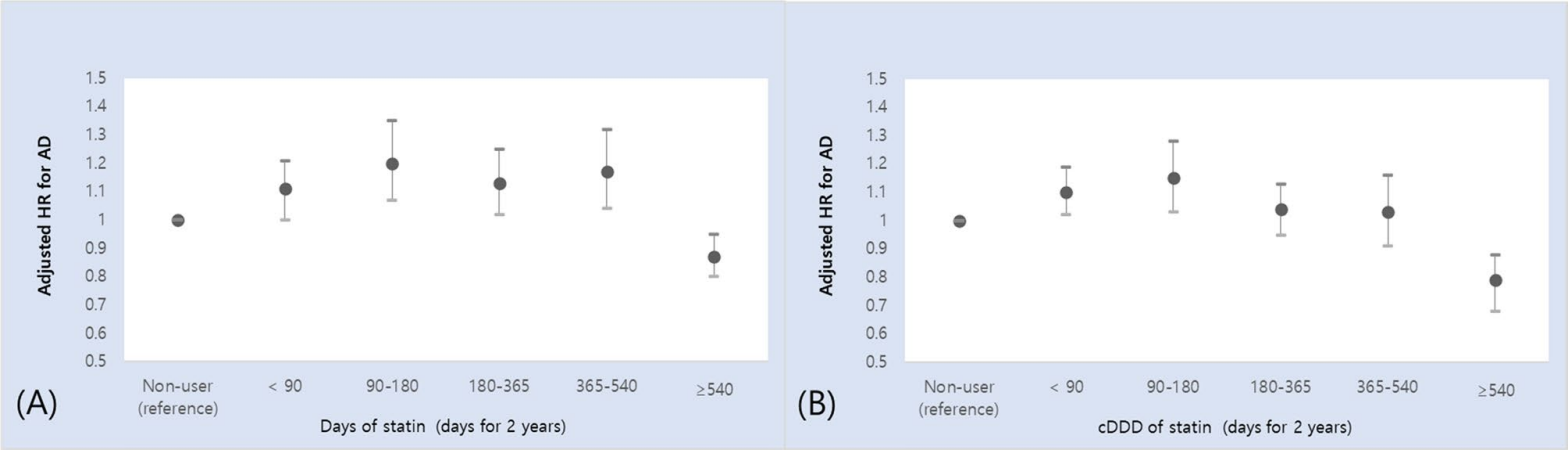
Association between statin use and incidence of Alzheimer’s disease (AD). Adjusted hazard ratios (HRs) for AD according to (**A**) duration of stain use and (**B**) cumulative defined daily dose (cDDD) for each 2-year period are shown. Statin use (≥ 540 days or cDDD) was associated with reduced risk of AD, while statin use (< 540 days or cDDD) was associated with increased risk of AD compared to statin non-use. Statin exposure by cDDD and incidence of AD showed an inverse J-shaped association with a significant P for trend among statin users (P-trend < 0.001).

The AD incidence during the follow-up period was 9,467 (8.0%) cases during a median follow-up of 7.2 years (Table 1). Overall, statin use itself was not significantly associated with AD incidence [adjusted HR (aHR) = 1.04, 95% confidence interval (CI) = 0.99–1.10] (Table 2). However, when examined by level of statin exposure, days of prescription for < 540 days during a 2-year period was associated with a higher risk of AD [aHR (95% CI) = 1.11 (1.00–1.21); for < 90 days, 1.20 (1.07–1.35); for 90–180 days, 1.13 (1.02–1.25); for 180–365 days, and 1.17 (1.04–1.32) for 365–540 days] compared to statin non-use. However, days of prescription ≥ 540 days was associated with a decreased risk of AD [aHR (95% CI) = 0.87 (0.80–0.95)]. Moreover, cDDD < 180 of statin use was significantly associated with an increased risk of AD [aHR (95% CI) = 1.10 (1.02–1.19); for < 90 days and 1.15 (1.03–1.28) in 90–180 days] compared to statin non-use. On the other hand, cDDD ≥ 540 was associated with a decreased risk of AD [aHR (95% CI) = 0.79 (0.68–0.92)]. Overall, statin exposure and incidence of AD had an inverse J-shaped association (Fig. 1). Analysis with further categorization showed further decreased risk of AD was observed in patients with ≥ 720 days of statin use [aHR (95% CI) = 0.76 (0.61–0.94)] and cDDD ≥ 900 [aHR (95% CI) = 0.35(0.22–0.55)], with a dose–response relationship (Supplementary Table 3). Additional adjustment for type of statin did not change the association of statin use with risk of AD, and no significant difference by type of statin (e.g., fungus-derived and synthetic) was observed for risk of AD (Supplementary Table 4).

**Table 2:** Association of statin use with incidence of Alzheimer’s disease.

	Unadjusted		Adjusted			
	HR (95% CI)	*P* value	Model 1		Model 2	
			aHR (95% CI)	*P* value	aHR (95% CI)	*P* value
**Statin use**	0.99 (0.95–1.05)	0.985	1.05 (1.00–1.11)	0.038	1.04 (0.99–1.10)	0.097
**Days of statin use (for 2 years)**						
Non-use	Reference		Reference		Reference	
< 90	1.06 (0.96–1.17)	0.234	1.11 (1.01–1.22)	0.039	1.11 (1.00–1.21)	0.045
90–180	1.12 (0.99–1.26)	0.053	1.20 (1.07–1.35)	0.002	1.20 (1.07–1.35)	0.002
180–365	1.04 (0.94–1.15)	0.421	1.13 (1.02–1.26)	0.016	1.13 (1.02–1.25)	0.023
365–540	1.11 (0.99–1.25)	0.079	1.18 (1.05–1.33)	0.005	1.17 (1.04–1.32)	0.009
≥ 540	0.86 (0.79–0.93)	< 0.001	0.89 (0.82–0.97)	0.005	0.87 (0.80–0.95)	0.001
P for trend	0.077		0.934		0.057	
**cDDD of statin use (for 2 years)**						
Non-use	Reference		Reference		Reference	
< 90	1.05 (0.97–1.14)	0.215	1.11 (1.02–1.20)	0.016	1.10 (1.02–1.19)	0.021
90–180	1.07 (0.96–1.19)	0.202	1.15 (1.03–1.28)	0.010	1.15 (1.03–1.28)	0.013
180–365	0.99 (0.91–1.08)	0.820	1.05 (0.97–1.14)	0.237	1.04 (0.95–1.13)	0.374
365–540	0.99 (0.89–1.13)	0.999	1.05 (0.93–1.18)	0.458	1.03 (0.91–1.16)	0.680
≥ 540	0.78 (0.67–0.90)	0.001	0.81 (0.69–0.93)	0.004	0.79 (0.68–0.92)	0.002
P for trend	0.109		0.928		0.066	

*cDDD* cumulative defined daily dose, *HR* hazard ratio, *aHR* adjusted hazard ratio, *CI* confidence interval.

Model 1 was adjusted for age and sex. Model 2 was adjusted for model 1 + body mass index, income, smoking status, alcohol status, hypertension, diabetes, and baseline cholesterol level.

## Discussion

We investigated the dose–response relationship between statin use and the incidence of AD while considering time-varying exposure. The strengths of our study include (1) a large population-based representative sample; (2) the use of pharmacy claims data that enabled us to collect accurate information on statin use including dosage and statin intensity; and (3) the use of time-dependent analysis that minimized immortal time bias^20^.

Statin use dramatically increased during our study period from 7.2% in 2002–2003 to 33.8% in 2010–2011. In addition, persistence also dramatically increased: 64.5% of statin users showed cDDD < 90 days in 2002–2003, but only 16.9% had a cDDD this low in 2010–2011. This marked increasing trend for statin prescriptions may be due to the publication of large randomized trials, adoption of new statins, and/or release of generic drugs^21^. This trend is similar in other countries and suggests that previous studies that do not consider statin use as a time-varying covariate suffer from misclassification bias.

Our study showed that statin users with poor persistence and poor adherence had an elevated risk for AD compared to statin non-users. While this seems to indicate that statin use can increase AD risk, we should consider indication bias. Statin treatment is indicated in patients with high cholesterol levels or at high risk of developing cardiovascular events. Conversely, statin non-users in our study were not at such risk. As hypercholesterolemia or comorbid cardiovascular risk factors are associated with higher AD risk^22^, an increased risk in the poor persistence and poor adherence group may be a reflection of such risk. In our study, both high and low cholesterol levels were also associated with higher risk of developing AD. Hypercholesterolemia has received attention as a risk factor for cognitive decline or AD^23,24^. High cholesterol levels in serum have been positively correlated with accumulation of amyloid β in the brains of patients with AD^25^.

On the contrary, patients who maintained their statin prescription with good adherence had an even lower risk of developing AD than statin non-users, suggesting a protective effect of statin use. The more evident dose–response relationship by cDDD compared to days of statin prescription suggests that this protective effect is also dependent upon statin dosage and potency. Our finding is consistent with previous studies that showed a stronger protective effect with a longer cumulative duration of statin use and use of a higher potency statin^18,26^.

There are multiple possible explanations for our findings, which may suggest either a causal relationship, confounding effect, or even reverse causal relationship. First, a direct protective effect of statin use on AD can be suggested. As statins are usually prescribed when hypercholesterolemia is detected, statin users with hypercholesterolemia are possibly at a higher risk of AD^27^. Our study findings suggest that persistent use with good compliance can reverse the AD risk, while an elevated risk cannot be reduced by non-persistent use. Our epidemiological finding is also supported by preclinical evidence. Experimental studies have suggested that simvastatin reduced the level of Aβ in the brain of guinea pigs^28^ and reduced levels of AD markers in cerebrospinal fluid in humans^29^. A brain autopsy study showed that patients taking statins were less likely to have amyloid deposits, although there was no relationship between statins and amyloid load^30^. Second, there is a possibility that non-persistent use of and poor adherence to statin are markers of a less healthy lifestyle or lower socioeconomic status, which are also AD risk factors. Heavy alcohol consumption^31^, and lower educational attainment^32^ are both reported to be associated with the occurrence of AD. On the contrary, good persistence and adherence to statin therapy could be a marker of a healthier lifestyle and socioeconomic status, rather than suggesting a direct effect of statin use on AD. Third, there is the possibility of an inverse association between statin use and AD. Poor drug adherence might be a sign of the prodromal stage of AD. Executive function and working memory are shown to be important factors for medication adherence^33^. Even very mild cognitive impairment can contribute to inadequate medication adherence^34^. However, the more evident association between cDDD and AD risk is not well addressed by inverse causality, suggesting that the first theory is a more plausible explanation of our findings.

Our study has important clinical and public health implications. Some people hesitate to take statins or discontinue their use due to concerns about long-term adverse effects such as dementia; however, individuals who are at high risk of cardiovascular disease should not be discouraged from taking statins due to concerns about cognitive impairment. In addition, our findings suggest that persistent statin use should be encouraged among patients who have begun taking statins because of the potential for decreased risk of both cardiovascular disease and AD.

There are several limitations that need to be considered when interpreting our study. First, there could be concerns about indication bias as people who are and are not treated with statins might differ in terms of cardiovascular condition, functional status, and health behaviors, which are often not measured. A randomized controlled trial would be the only solution to overcome this bias, but is not feasible due to practical and ethical concerns. In this observational study, we tried to overcome this by investigating dose–response relationship by adjustment of all potential confounders available in our data. In addition, we conducted sensitivity analyses with a subgroup of participants who were indicated for statin treatment (Supplementary Table 5), using propensity score matching or adjustment (Supplementary Table 6), and the results were consistent with the main analyses. Second, as the national data were not intentionally collected solely for this study, we could not obtain information on AD-related factors, such as education level and *APOE* genotype. Third, our outcome determination is based on claims data from clinics and hospitals based on a diagnosis of AD and prescription of an antidementia drug. Underuse of antidementia drugs might cause selection bias in the definition of AD incidence. As a periodic screening test for AD was not conducted as in a prospective cohort study, there is the possibility of underdiagnosis, especially in individuals with poor medical care access. However, this suggests that the protective effect of statin use on AD could be even stronger. Fourth, the subtypes of dementia cannot be clearly distinguished using claims data, and accurate determination of subtype is not always performed in a typical primary care practice where brain magnetic resonance imaging is often not available^35^. In addition, AD and vascular dementia frequently co-exist, which accounts for the protective effect of statin therapy on AD^36^. However, after excluding AD cases with vascular dementia, the results were not changed (Supplementary Table 7). Fifth, we did not consider concomitant drugs as potential confounders in our analysis. Currently, several medications, such as metformin^37^, aspirin^38^, benzodiazepines^39^, and zolpidem^40^ are suspected to affect cognitive function and dementia incidence. However, the evidence is too conflicting^41,42^ to be included as definite confounders. Sixth, reverse causality could exist. Preclinical or early dementia (before it is formally diagnosed) increases the risk of treatment noncompliance. To complement this possibility, we additionally performed 2-year, 4-year, 6-year, and 8-year lag time analysis, excluding dementia cases that occurred in the time lags. The results at 2-year and 4-year lags were consistent with the main findings, although the confidence interval became wider and the significance level was attenuated (Supplementary Table 8). However, this association was not evident with longer lag times (6 or 8 years) probably due to the relatively short follow up in our study. Finally, although factors for adherence include age, socioeconomic status, social support, depression, and co-payments and could confound our results, these factors were not considered fully in our study due to lack of information. Our findings indicate that, although hypercholesterolemia can increase the risk of AD, persistent and adherent use of statins can reduce and even reverse the risk. Statin use itself is not associated with AD risk, and persistence of and good adherence to statin therapy should be emphasized in clinical practice and public health education.

## Methods

### Study setting

The National Health Insurance (NHI) in Korea covers approximately 97% of the Korean population, while the remaining 3% are covered by the Medical Aid program. All medical utilization and prescription information covered by NHI are collected with International Classification of Diseases, 10th revision (ICD-10) diagnosis codes for each outpatient visit or hospitalization, since medical facilities and pharmacies submit medical and pharmacy claims for reimbursement.

A biennial national health screening program (NHSP) is provided to all NHI members over 40 years of age, which contains a questionnaire on health behavior (e.g., past medical history, smoking, and alcohol consumption), anthropometric measurements (e.g., weight, height, and blood pressure), and a blood test (e.g., fasting glucose and lipid levels) to screen for cardiovascular risk factors. In addition, NHI has established a national database linked to the results of the NHSP, including information on utilization of medical facilities and a death registry database^43^. This national database is widely used in epidemiologic studies^44^.

This study was approved by Samsung Medical Center’s Institutional Review Board (IRB number: SMC 2017-05-131), and consent from individual patients was waived as the data is public and anonymized under confidentiality guidelines. The study was conducted according to the tenets of the Declaration of Helsinki and followed the STROBE (Strengthening the Reporting of Observational Studies in Epidemiology) guidelines.

### Study population

This study used the National Health Insurance Service (NHIS)-National Health Screening Cohort (HEALS) database, which consists of information on 514,866 Koreans 40–79 years old who were in the NHSP from 2002–2003. They were followed-up until 31 December 2013. Among the 514,866 Koreans in the database, we identified 132,853 subjects over 60 years old. We excluded subjects who died (n = 862), and those who had experienced dementia (n = 282) or stroke (n = 6735) before the index date (1 January 2004), and those who had missing information (n = 5961) at baseline, leaving a final study population of 119,013 subjects free of dementia and stroke. Subjects with an ICD-10 diagnosis of dementia (F00–F03, F05, G30, and G31) or stroke (I60-I69) based on claim data for NHI were excluded.

### Exposure: assessment of statin use

NHI prescription records include the date, dosage, duration, and generic names for all medications. To reflect changes in the use of statin over time (e.g., new initiation or discontinuation, change to other statins), statin use was defined as a time-varying variable for every 2-year time window from 2002–2003^45^. To reflect cumulative exposure to statins, we used two definitions for statin exposure: days of statin prescription and cumulative defined daily dose (cDDD) during each 2-year time window. Days of statin prescription was defined as the total number of days of statin prescription regardless of dosage or intensity. The cDDD was defined as the summation of total DDD and was considered not only the number of prescription days, but also the dosage and therapeutic intensity of the drug.

To examine the dose–response relationship, we categorized days of statin prescription and cDDD into five groups: < 90, 90–180, 180–365, 365–540, and ≥ 540 days and cDDD considering the distribution of statin use. Based on this definition, a larger number of days of statin prescription and cDDD can be regarded as markers of persistent and adherent use of statin therapy (which means ‘not discontinuing’ and ‘not skipping’ the medication, respectively).

### Outcome: incidence of dementia

The incidence of AD was defined using the prescription records for anti-Alzheimer drugs (donepezil, galantamine, rivastigmine, or memantine) with ICD-10 codes related to AD (F00 and G30) in medical expense claims submitted to the NHI service^44^. In Korea, there is a relatively strict requirement to be reimbursed for prescriptions for anti-Alzheimer drugs if a patient has a Mini Mental State Examination score ≤ 26 and either a Clinical Dementia Rating ≥ 1 or a Global Deterioration Scale score ≥ 3. Physicians who prescribe these anti-Alzheimer drugs need to document the evidence in patients’ medical records in accordance with NHI’s strict reimbursement policy.

### Statistical analysis

We used the t-test and chi-square tests for continuous and categorical variables to examine the differences between statin non-users and statin users. Time-dependent Cox proportional hazards regression analyses were performed to evaluate the association between statin use and the incidence of AD. The time-dependent model has strengths over the conventional Cox model in that it is able to reflect the time-varying factors over time rather than as fixed baseline factors^46^. The follow-up time for each subject was divided into a time window of 2 years, and the cumulative use of each medication during the previous time window (i.e., previous 2 years) was calculated as exposure (Supplementary Fig. 1). This was also used in previous studies examining the effect of statin use on dementia^15,17^ or medication adherence on mortality^47^. The statin use, days of statin prescription, and cDDD were modeled as time-dependent variables. We adjusted for age and sex in model 1. In addition, we adjusted for lifestyle variables (BMI, smoking status, and alcohol consumption), socioeconomic factors (income status), and medical information (history of hypertension, diabetes, and serum cholesterol levels) in model 2.

In sensitivity analyses, (1) we restricted inclusion to a cohort for whom statins were indicated (n = 34,526), including patients with diabetes, those who had high total cholesterol ≥ 240 mg/dL, and inpatient or outpatient diagnosis history of cardiovascular events (I21, I22, I60, I61, I62, I63, I64), (2) we performed a propensity score matching (1:3) analysis with adjustment for propensity scores to determine the independence of association between statin use and AD, and (3) we applied 2-year, 4-year, 6-year, and 8-year lag times to examine reverse causality. All statistical analyses were carried out using Stata version 14.1 (Stata Corp, College Station, TX, USA).

## Supplementary Information

Supplementary Information.
