# Supplementary material for: Association between statin use and Alzheimer’s disease with dose response relationship
Source: Sci Rep. 2021 Jul 27;11:15280. doi: 10.1038/s41598-021-94803-3 (PMC8316580; doi:10.1038/s41598-021-94803-3)

**Association between statin use and Alzheimer’s disease with dose response relationship**

**Running head: Statin and Alzheimer’s disease**

**Authors**: Su-Min Jeong, MD.^1,2^; Dong Wook Shin, MD., DrPH.; MBA.^3,4^, Tae Gon Yoo, MD., MPH^5^; Mi Hee Cho, MD., PhD.^6^; Wooyoung Jang, MD., PhD.^7^; Jinkook Lee, PhD.^8^; SangYun Kim, MD., PhD.^9^

**Affiliations:**

^1^Department of Family Medicine, Seoul Metropolitan Government-Seoul National University Boramae Medical Center & Seoul National University Health Service Center Seoul South Korea

^2^ Harvard T.H. Chan School of Public Health, Department of Nutrition, Boston, MA, USA

^3^ Department of Family Medicine/Supportive Care Center, Samsung Medical Center

^4^ Department of Clinical Research Design & Evaluation, Samsung Advanced Institute for Health Science & Technology (SAIHST), Sungkyunkwan University

^5^ Department of Family Medicine, Hongseong Medical Center, Hongseong, Republic of Korea

^6^ Samsung C&T Medical Clinic, Kangbuk Samsung Hospital, Seoul, Republic of Korea

^7^ Department of Neurology, Gangneung Asan Hospital, University of Ulsan College of Medicine, Gangneung, Republic of Korea

^8^ University of Southern California, Department of Economics & Center for Economic & Social Research, Los Angeles, & RANC Corporation, Santa Monica, CA, USA

^9^ Seoul National University Bundang Hospital & Seoul National University College of Medicine, Department of Neurology, Seongnam, Republic of Korea

**Supplementary materials**

**Supplementary Table 1. Baseline characteristics of the study population during 2002-2003**

|  |  | | Total  (N = 119,013) | | Non-statin user  (N = 110,392) | | Statin user  (N = 8,621) | *p* value |
| --- | --- | --- | --- | --- | --- | --- | --- | --- |
| Age, years, mean (SD) | | 66.0 (4.9) | | 66.1 (4.9) | | 65.7 (4.5) | | < 0.001 |
| Sex, n (%) | |  | |  | |  | | < 0.001 |
| Men | | 57,240 (48.1) | | 54,279 (49.2) | | 2,961 (34.4) | |  |
| Women | | 61,773 (51.9) | | 56,113 (50.8) | | 5,660 (65.6) | |  |
| Body mass index, kg/m^2^, mean (SD) | | 23.8 (3.2) | | 23.7 (3.1) | | 25.1 (3.1) | | < 0.001 |
| Total cholesterol, mg/dL, mean (SD) | | 202.2 (39.5) | | 200.4 (38.2) | | 225.1 (48.0) | | < 0.001 |
| Household income, n (%) | |  | |  | |  | | < 0.001 |
| High | | 45,460 (38.2) | | 41,498 (37.6) | | 3,962 (46.0) | |  |
| Middle | | 121,883 (25.3) | | 27,243 (24.7) | | 1,989 (23.0) | |  |
| Low or Medicaid | | 145,787 (30.4) | | 41,651 (37.7) | | 2 670 (31.0) | |  |
| Smoking status, n (%) | |  | |  | |  | | < 0.001 |
| Never | | 89,275 (75.0) | | 82,150 (74.4) | | 7,125 (82.7) | |  |
| Former | | 8,645 (7.3) | | 8,074 (7.3) | | 571 (6.6) | |  |
| Current | | 21,093 (17.7) | | 20,168 (18.3) | | 925 (10.7) | |  |
| Alcohol consumption, frequency, n (%) | |  | |  | |  | | < 0.001 |
| None | | 82,736 (69.5) | | 76,079 (68.9) | | 6,657 (77.2) | |  |
| 2-3 monthly | | 10,942 (9.2) | | 10,229 (9.3) | | 713 (8.3) | |  |
| 1-2 weekly | | 11,270 (9.5) | | 10,623 (9.6) | | 647 (7.5) | |  |
| 3-4 weekly | | 6,276 (5.3) | | 5,949 (5.4) | | 327 (3.8) | |  |
| Almost every day | | 7,789 (6.5) | | 7,512 (6.8) | | 277 (3.2) | |  |
| Hypertension, n (%) | |  | |  | |  | | < 0.001 |
| No | | 57,643 (48.4) | | 54,313 (49.2) | | 3,330 (38.6) | |  |
| Yes | | 61,370 (51.6) | | 56,079 (50.8) | | 5,291 (61.4) | |  |
| Diabetes, n (%) | |  | |  | |  | | < 0.001 |
| No | | 101,206 (85.0) | | 94,812 (85.9) | | 6,394 (74.2) | |  |
| Yes | | 17,807 (15.0) | | 15,580 (14.1) | | 2,227 (25.8) | |  |

SD, standard deviation

**Supplementary Table 2. Association between cholesterol level and risk of Alzheimer’s disease**

| **Total cholesterol, mg/dL** | **N (%)** | Unadjusted HR (95% CI) | Adjusted HR (95% CI) | |
| --- | --- | --- | --- | --- |
|  |  |  | Model 1 | Model 2* |
| Total |  |  |  |  |
| < 160 | 15,344 (12.9) | 1.10 (1.03 – 1.18) | 1.15 (1.07 – 1.22) | 1.12 (1.05 – 1.20) |
| 160 – 200 | 43,858 (36.9) | 0.98 (0.94 – 1.03) | 0.99 (0.95 – 1.05) | 0.99 (0.94 – 1.04) |
| 200 – 240 | 40,515 (34.0) | Reference | Reference | Reference |
| 240 – 300 | 17,556 (14.8) | 1.09 (1.03 – 1.16) | 1.05 (0.99 – 1.12) | 1.06 (0.99 – 1.12) |
| ≥ 300 | 1,740 (1.5) | 1.31 (1.13 – 1.53) | 1.22 (1.05 – 1.42) | 1.22 (1.05 – 1.42) |
| Without statin use |  |  |  |  |
| < 160 | 14,914 (12.9) | 1.10 (1.03 – 1.17) | 1.14 (1.06 – 1.22) | 1.11 (1.04 – 1.19) |
| 160 – 200 | 42,909 (37.0) | 0.98 (0.94 – 1.03) | 1.00 (0.95 – 1.05) | 0.99 (0.94 – 1.04) |
| 200 – 240 | 39,675 (34.2) | Reference | Reference | Reference |
| 240 – 300 | 16,887 (14.6) | 1.09 (1.02 – 1.16) | 1.05 (0.99 – 1.12) | 1.05 (0.99 – 1.12) |
| ≥ 300 | 1,608 (1.4) | 1.34 (1.15 – 1.57) | 1.25 (1.07 – 1.46) | 1.25 (1.07 – 1.45) |
| With statin use |  |  |  |  |
| < 160 | 430 (14.2) | 1.42 (0.96 – 2.10) | 1.42 (0.96 – 2.11) | 1.37 (0.92 – 2.04) |
| 160 – 200 | 949 (31.4) | 1.12 (0.80 – 1.57) | 1.13 (0.80 – 1.59) | 1.14 (0.81 – 1.60) |
| 200 – 240 | 840 (27.8) | Reference | Reference | Reference |
| 240 – 300 | 669 (22.2) | 1.24 (0.87 – 1.78) | 1.20 (0.84 – 1.71) | 1.23 (0.86 – 1.76) |
| ≥ 300 | 132 (4.4) | 1.04 (0.53 – 2.02) | 0.95 (0.49 – 1.86) | 0.95 (0.48 – 1.86) |

Model 1 was adjusted for age and sex.

* Model 2 was adjusted for model 1 + body mass index, income, smoking status, alcohol status, hypertension, diabetes and model 2 in total was additionally adjusted for statin use.

**Supplementary Table 3. Association of statin use with incidence of Alzheimer’s disease with further categorization**

|  | **Unadjusted**  HR (95% CI) |  | **Adjusted** | | | |
| --- | --- | --- | --- | --- | --- | --- |
|  |  |  | Model 1 | | Model 2 | |
|  |  | *P* value | aHR (95% CI) | *P* value | aHR (95% CI) | *P* value |
| **Days of statin use**  **(for 2 years)** |  |  |  |  |  |  |
| Non-use | Reference |  | Reference |  | Reference |  |
| < 90 | 1.06 (0.96 – 1.17) | 0.234 | 1.11 (1.01 – 1.22) | 0.039 | 1.11 (1.00 – 1.21) | 0.045 |
| 90 – 180 | 1.12 (0.99 – 1.26) | 0.053 | 1.20 (1.07 – 1.35) | 0.002 | 1.20 (1.07 – 1.35) | 0.002 |
| 180 – 365 | 1.04 (0.94 – 1.15) | 0.421 | 1.13 (1.02 – 1.26) | 0.016 | 1.13 (1.02 – 1.25) | 0.023 |
| 365 – 540 | 1.11 (0.99 – 1.25) | 0.079 | 1.18 (1.05 – 1.33) | 0.005 | 1.17 (1.04 – 1.32) | 0.009 |
| 540 – 720 | 0.87 (0.80 - 0.95) | 0.002 | 0.91 (0.83 – 0.99) | 0.033 | 0.89 (0.82 – 0.98) | 0.013 |
| ≥ 720 | 0.77 (0.62 - 0.96) | 0.018 | 0.77 (0.62 -0.96) | 0.021 | 0.76 (0.61 -0.94) | 0.012 |
| P for trend | 0.059 |  | 0.803 |  | 0.403 |  |
| **cDDD of statin use**  **(for 2 years)** |  |  |  |  |  |  |
| Non-use | Reference |  | Reference |  | Reference |  |
| < 90 | 1.05 (0.97 – 1.14) | 0.215 | 1.11 (1.02 – 1.20) | 0.016 | 1.10 (1.02 – 1.19) | 0.021 |
| 90 – 180 | 1.07 (0.96 – 1.19) | 0.202 | 1.15 (1.03 – 1.28) | 0.010 | 1.15 (1.03 – 1.28) | 0.013 |
| 180 – 365 | 0.99 (0.91 – 1.08) | 0.820 | 1.05 (0.97 – 1.14) | 0.237 | 1.04 (0.95 – 1.13) | 0.374 |
| 365 – 540 | 1.00 (0.89 – 1.13) | 0.999 | 1.05 (0.93 – 1.18) | 0.458 | 1.03 (0.91 – 1.16) | 0.680 |
| 540 – 720 | 0.90 (0.75 – 1.07) | 0.220 | 0.93 (0.78 - 1.11) | 0.445 | 0.92 (0.77 – 1.09) | 0.326 |
| 720 – 900 | 0.97 (0.69 - 1.36) | 0.869 | 1.01 (0.72 – 1.41) | 0.964 | 0.99 (0.70 – 1.38) | 0.933 |
| ≥ 900 | 0.35 (0.22 – 0.54) | < 0.001 | 0.35 (0.22 – 0.55) | < 0.001 | 0.35 (0.22 – 0.55) | < 0.001 |
| P for trend | 0.036 |  | 0.630 |  | 0.290 |  |

cDDD, cumulative defined daily dose; HR, hazard ratio; aHR, adjusted hazard ratio; CI, confidence interval.

Model 1 was adjusted for age and sex. Model 2 was adjusted for model 1 + body mass index, income, smoking status, alcohol status, hypertension, diabetes, and baseline cholesterol level

**Supplementary Table 4. Association of statin types with incidence of Alzheimer’s disease among statin users over follow-up period (n = 40,521)**

|  | Model 1 | | Model 2 | | Model 3 | |
| --- | --- | --- | --- | --- | --- | --- |
|  | HR (95% CI) | *P* value | aHR (95% CI) | *P* value | aHR (95% CI) | *P* value |
| **Type of statins use*** |  |  |  |  |  |  |
| Fungus-derived statin | Reference |  | Reference |  | Reference |  |
| Synthetic statin | 0.97 (0.87 – 1.07) | 0.485 | 0.97 (0.88 – 1.07) | 0.586 | 0.97 (0.88 – 1.07) | 0.563 |
| Mixed use | 1.17 (1.03 – 1.34) | 0.015 | 1.18 (1.04 – 1.35) | 0.010 | 1.18 (1.04 – 1.35) | 0.010 |
| **Days of statin use** |  |  |  |  |  |  |
| < 90 | Reference |  | Reference |  | Reference |  |
| 90 – 180 | 1.04 (0.90 – 1.22) | 0.528 | 1.07 (0.92 – 1.24) | 0.358 | 1.07 (0.93 - 1.25) | 0.348 |
| 180 – 365 | 0.97 (0.85 – 1.12) | 0.681 | 1.00 (0.97 – 1.15) | 0.953 | 1.01 (0.88 - 1.16) | 0.929 |
| 365 – 540 | 1.03 (0.89 – 1.20) | 0.700 | 1.04 (0.90 – 1.21) | 0.583 | 1.05 (0.90 – 1.22) | 0.572 |
| ≥ 540 | 0.85 (0.75 – 0.96) | 0.009 | 0.84 (0.74 – 0.95) | 0.005 | 0.83 (0.73 - 0.94) | 0.003 |
| **Type of statins use*** |  |  |  |  |  |  |
| Fungus-derived statin | Reference |  | Reference |  | Reference |  |
| Synthetic statin | 0.97 (0.88 – 1.08) | 0.574 | 0.98 (0.88 – 1.08) | 0.680 | 0.98 (0.88 – 1.08) | 0.656 |
| Mixed use | 1.18 (1.04 – 1.34) | 0.012 | 1.19 (1.05 – 1.35) | 0.008 | 1.19 (1.05 – 1.35) | 0.008 |
| **cDDD of statin use** |  |  |  |  |  |  |
| < 90 | Reference |  | Reference |  | Reference |  |
| 90 – 180 | 1.01 (0.89 – 1.16) | 0.850 | 1.03 (0.90 – 1.17) | 0.665 | 1.03 (0.91 - 1.18) | 0.631 |
| 180 – 365 | 0.94 (0.84 – 1.06) | 0.306 | 0.95 (0.84 – 1.06) | 0.333 | 0.94 (0.84 - 1.06) | 0.322 |
| 365 – 540 | 0.93 (0.81 – 1.08) | 0.346 | 0.93 (0.80 – 1.07) | 0.299 | 0.92 (0.79 - 1.06) | 0.247 |
| ≥ 540 | 0.87 (0.74 – 1.03) | 0.117 | 0.86 (0.73 – 1.02) | 0.087 | 0.85 (0.72 – 1.01) | 0.067 |

cDDD, cumulative defined daily dose; HR, hazard ratio; aHR, adjusted hazard ratio; CI, confidence interval.

* Fungus-derived statins include simvastatin, lovastatin, and pravastatin and synthetic statins include atorvastatin, fluvastatin, rosuvastatin, and pitavastatin.

Model 1 was mutually adjusted for type of statin and days of statin use or type of statin and cDDD of statin use.

Model 2 was additionally adjusted for model 1 + age and sex

Model 3 was additionally adjusted for model 1 + body mass index, income, smoking status, alcohol status, hypertension, diabetes, and baseline cholesterol level.

**Supplementary Table 5. Association of statin use with incidence of Alzheimer’s disease among subgroup of participants who were indicated for statin therapy (n =34,526)**

|  | **Unadjusted**  HR (95% CI) |  | **Adjusted** | | | |
| --- | --- | --- | --- | --- | --- | --- |
|  |  |  | Model 1 | | Model 2 | |
|  |  | *P* value | aHR (95% CI) | *P* value | aHR (95% CI) | *P* value |
| **Statin use** | 0.89 (0.83 - 0.96) | 0.003 | 0.97 (0.90 - 1.04) | 0.397 | 1.00 (0.92 - 1.08) | 0.998 |
| **Days of statin use (for 2 years)** |  |  |  |  |  |  |
| Non-use | Reference |  | Reference |  | Reference |  |
| < 90 | 0.91 (0.79 - 1.06) | 0.244 | 0.97 (0.84 - 1.13) | 0.737 | 1.02 (0.87 - 1.18) | 0.837 |
| 90 – 180 | 1.04 (0.88 - 1.23) | 0.606 | 1.14 (0.96 - 1.34) | 0.131 | 1.19 (1.00 - 1.41) | 0.044 |
| 180 – 365 | 0.92 (0.79 - 1.07) | 0.264 | 1.03 (0.89 - 1.20) | 0.696 | 1.07 (0.92 - 1.24) | 0.380 |
| 365 – 540 | 1.02 (0.87 - 1.20) | 0.831 | 1.13 (0.96 - 1.33) | 0.135 | 1.17 (1.00 - 1.38) | 0.054 |
| ≥ 540 | 0.77 (0.68 - 0.86) | <0.001 | 0.82 (0.73 - 0.93) | 0.001 | 0.84 (0.75 - 0.95) | 0.004 |
| *P* for trend | < 0.001 |  | 0.055 |  | 0.185 |  |
| **cDDD of statin use (for 2 years)** |  |  |  |  |  |  |
| Non-use | Reference |  | Reference |  | Reference |  |
| < 90 | 0.93 (0.82 - 1.05) | 0.240 | 0.99 (0.87 - 1.12) | 0.837 | 1.03 (0.91 - 1.17) | 0.676 |
| 90 – 180 | 1.03 (0.88 - 1.19) | 0.743 | 1.14 (0.98 - 1.33) | 0.083 | 1.19 (1.02 - 1.39) | 0.026 |
| 180 – 365 | 0.86 (0.76 - 0.97) | 0.014 | 0.95 (0.84 - 1.07) | 0.405 | 0.98 (0.87 - 1.11) | 0.746 |
| 365 – 540 | 0.88 (0.74 - 1.03) | 0.121 | 0.94 (0.80 - 1.11) | 0.487 | 0.97 (0.82 - 1.14) | 0.687 |
| ≥ 540 | 0.71 (0.58 - 0.88) | 0.001 | 0.77 (0.63 - 0.94) | 0.012 | 0.79 (0.64 - 0.97) | 0.022 |
| *P* for trend | < 0.001 |  | 0.063 |  | 0.202 |  |

cDDD, cumulative defined daily dose; HR, hazard ratio; aHR, adjusted hazard ratio; CI, confidence interval.

Model 1 was adjusted for age and sex. Model 2 was adjusted for model 1 + body mass index, income, smoking status, alcohol status, hypertension, diabetes, and baseline cholesterol level

**Supplementary Table 6. Association of statin use with incidence of Alzheimer’s disease with propensity score methods**

|  | **Matched on propensity score (1:3)*** | | **Adjusted for propensity score** | |
| --- | --- | --- | --- | --- |
|  | aHR (95% CI) | *P* value | aHR (95% CI) | *P* value |
| **Statin use** | 1.01 (0.93 - 1.09) | 0.838 | 1.04 (0.99 - 1.10) | 0.122 |
| **Days of statin use**  **(for 2 years)** |  |  |  |  |
| Non-use | Reference |  | Reference |  |
| < 90 | 1.06 (0.90 - 1.24) | 0.487 | 1.10 (1.00 - 1.22) | 0.048 |
| 90 – 180 | 1.27 (1.07 - 1.51) | 0.006 | 1.20 (1.06 - 1.34) | 0.003 |
| 180 – 365 | 1.05 (0.90 - 1.22) | 0.573 | 1.12 (1.01 - 1.25) | 0.027 |
| 365 – 540 | 1.13 (0.95 - 1.34) | 0.160 | 1.17 (1.04 - 1.31) | 0.011 |
| ≥ 540 | 0.86 (0.76 - 0.96) | 0.010 | 0.87 (0.80 - 0.95) | 0.001 |
| P for trend | 0.176 |  | 0.437 |  |
| **cDDD of statin use**  **(for 2 years)** |  |  |  |  |
| Non-use | Reference |  | Reference |  |
| < 90 | 1.09 (0.96 - 1.24) | 0.190 | 1.10 (1.01 - 1.20) | 0.023 |
| 90 – 180 | 1.11 (0.94 - 1.30) | 0.212 | 1.14 (1.03 - 1.28) | 0.015 |
| 180 – 365 | 0.98 (0.87 - 1.11) | 0.774 | 1.04 (0.95 - 1.13) | 0.419 |
| 365 – 540 | 0.99 (0.84 - 1.17) | 0.910 | 1.02 (0.90 - 1.15) | 0.727 |
| ≥ 540 | 0.79 (0.64 - 0.97) | 0.027 | 0.79 (0.68 - 0.92) | 0.002 |
| P for trend | 0.224 |  | 0.055 |  |

* 85,367 participants were included (Number of statin users = 8,440)

cDDD, cumulative defined daily dose; HR, hazard ratio; aHR, adjusted hazard ratio; CI, confidence interval.

aHR was adjusted for age, sex, body mass index, income, smoking status, alcohol status, hypertension, diabetes, and baseline cholesterol level

**Supplementary Table 7. Association of statin use with incidence of all dementia and Alzheimer’s disease after excluding vascular dementia**

|  | **Unadjusted**  HR (95% CI) |  | **Adjusted** | | | |
| --- | --- | --- | --- | --- | --- | --- |
|  |  |  | Model 1 | | Model 2 | |
|  |  | *P* value | aHR (95% CI) | *P* value | aHR (95% CI) | *P* value |
| **All dementia** |  |  |  |  |  |  |
| **Statin use** | 1.01 (0.96 – 1.05) | 0.817 | 1.06 (1.02 – 1.11) | 0.009 | 1.06 (1.01 – 1.11) | 0.027 |
| **Days of statin use**  **(for 2 years)** |  |  |  |  |  |  |
| Non-use | Reference |  | Reference |  | Reference |  |
| < 90 | 1.09 (0.99 – 1.19) | 0.069 | 1.14 (1.04 – 1.25) | 0.004 | 1.14 (1.04 – 1.25) | 0.004 |
| 90 – 180 | 1.14 (1.02 – 1.26) | 0.021 | 1.22 (1.01 – 1.23) | < 0.001 | 1.22 (1.09 – 1.36) | < 0.001 |
| 180 – 365 | 1.04 (0.95 – 1.15) | 0.401 | 1.14 (1.13 – 1.39) | 0.008 | 1.13 (1.03 – 1.25) | 0.011 |
| 365 – 540 | 1.12 (1.01 – 1.25) | 0.037 | 1.20 (1.08 – 1.34) | 0.001 | 1.19 (1.07 – 1.33) | 0.002 |
| ≥ 540 | 0.86 (0.79 – 0.92) | < 0.001 | 0.89 (0.82 – 0.96) | 0.002 | 0.87 (0.81 – 0.94) | < 0.001 |
| **cDDD of statin use**  **(for 2 years)** |  |  |  |  |  |  |
| Non-use | Reference |  | Reference |  | Reference |  |
| < 90 | 1.07 (0.99 – 1.16) | 0.070 | 1.13 (1.05 – 1.22) | 0.002 | 1.13 (1.05 – 1.22) | 0.002 |
| 90 – 180 | 1.07 (0.97 – 1.18) | 0.189 | 1.15 (1.04 – 1.27) | 0.005 | 1.15 (1.04 – 1.27) | 0.006 |
| 180 – 365 | 1.01 (0.94 – 1.09) | 0.700 | 1.08 (1.00 – 1.17) | 0.042 | 1.07 (0.99 – 1.16) | 0.085 |
| 365 – 540 | 0.98 (0.87 – 1.09) | 0.673 | 1.02 (0.92 – 1.14) | 0.684 | 1.00 (0.90 – 1.12) | 0.949 |
| ≥ 540 | 0.76 (0.66 – 0.86) | < 0.001 | 0.78 (0.67 – 0.89) | < 0.001 | 0.77 (0.67 – 0.88) | < 0.001 |
| **Alzheimer’s disease**  **(after excluding vascular dementia cases, n=1,298)** | |  |  |  |  |  |
| **Statin use** | 0.99 (0.95 – 1.05) | 0.750 | 1.05 (0.99 – 1.11) | 0.086 | 1.05 (0.99 – 1.11) | 0.100 |
| **Days of statin use**  **(for 2 years)** |  |  |  |  |  |  |
| Non-use | Reference |  | Reference |  | Reference |  |
| < 90 | 1.05 (0.95 – 1.17) | 0.355 | 1.10 (0.99 – 1.22) | 0.078 | 1.10 (0.99 – 1.23) | 0.067 |
| 90 – 180 | 1.11 (0.98 – 1.26) | 0.102 | 1.19 (1.05 – 1.35) | 0.006 | 1.20 (1.06 – 1.36) | 0.005 |
| 180 – 365 | 1.03 (0.92 – 1.15) | 0.660 | 1.12 (1.00 – 1.25) | 0.047 | 1.12 (1.00 – 1.25) | 0.044 |
| 365 – 540 | 1.12 (0.99 – 1.27) | 0.079 | 1.20 (1.05 – 1.35) | 0.005 | 1.19 (1.05 – 1.36) | 0.006 |
| ≥ 540 | 0.85 (0.78 – 0.93) | < 0.001 | 0.89 (0.81 – 0.97) | 0.007 | 0.88 (0.81 – 0.96) | 0.005 |
| **cDDD of statin use**  **(for 2 years)** |  |  |  |  |  |  |
| Non-use | Reference |  | Reference |  | Reference |  |
| < 90 | 1.05 (0.96 – 1.15) | 0.274 | 1.11 (1.01 – 1.21) | 0.027 | 1.11 (1.01 – 1.21) | 0.023 |
| 90 – 180 | 1.05 (0.93 – 1.18) | 0.428 | 1.13 (1.04 – 1.27) | 0.041 | 1.13 (1.01 – 1.28) | 0.035 |
| 180 – 365 | 0.99 (0.91 – 1.08) | 0.875 | 1.06 (0.97 – 1.15) | 0.221 | 1.05 (0.96 – 1.15) | 0.244 |
| 365 – 540 | 0.99 (0.87 – 1.13) | 0.887 | 1.04 (0.91 – 1.18) | 0.559 | 1.03 (0.90 – 1.17) | 0.654 |
| ≥ 540 | 0.76 (0.65 – 0.89) | 0.001 | 0.79 (0.67 – 0.92) | 0.003 | 0.78 (0.66 – 0.91) | 0.002 |

HR, hazard ratio; aHR, adjusted hazard ratio; CI, confidence interval.

Model 1 was adjusted for age and sex. Model 2 was adjusted for model 1 + body mass index, income, smoking status, alcohol status, hypertension, diabetes, and baseline cholesterol level

**Supplementary Table 8. Association of statin use with incidence of Alzheimer’s disease with lag times (2-year, 4-year, 6-year, and 8-year)**

|  | 2-year lag time |  | 4-year lag time |  | 6-year lag time |  | 8-year lag time |  |
| --- | --- | --- | --- | --- | --- | --- | --- | --- |
|  | aHR (95% CI) | *P* value | aHR (95% CI) | *P* value | aHR (95% CI) | *P* value | aHR (95% CI) | *P* value |
| **Statin use** | 1.01 (0.95 – 1.07) | 0.713 | 1.01(0.95 – 1.08) | 0.694 | 0.99 (0.91 – 1.08) | 0.776 | 1.06 (0.93 – 1.20) | 0.396 |
| **Days of statin use (for 2 years)** |  |  |  |  |  |  |  |  |
| Non-use | Reference |  | Reference |  | Reference |  | Reference |  |
| < 90 | 1.16 (1.05– 1.28) | 0.003 | 1.13 (1.02 – 1.26) | 0.021 | 1.07 (0.94 – 1.22) | 0.281 | 1.18 (0.98 – 1.41) | 0.076 |
| 90 – 180 | 0.94 (0.82 – 1.07) | 0.339 | 1.02 (0.88 – 1.19) | 0.750 | 0.86 (0.71 – 1.05) | 0.133 | 1.09 (0.84 – 1.43) | 0.508 |
| 180 – 365 | 1.00 (0.88 – 1.12) | 0.937 | 1.07 (0.94 – 1.22) | 0.323 | 0.98 (0.82 – 1.17) | 0.833 | 0.90 (0.67 – 1.22) | 0.505 |
| 365 – 540 | 1.15 (1.00 – 1.31) | 0.048 | 0.91 (0.76 – 1.10) | 0.326 | 1.00 (0.78 – 1.29) | 0.991 | 1.03 (0.68 – 1.58) | 0.873 |
| ≥ 540 | 0.87 (0.79 – 0.97) | 0.012 | 0.83 (0.72 – 0.96) | 0.015 | 0.94 (0.75 – 1.17) | 0.558 | 0.80 (0.51 – 1.24) | 0.309 |
| P for trend | 0.217 |  | 0.135 |  | 0.470 |  | 0.762 |  |
| **cDDD of statin use (for 2 years)** |  |  |  |  |  |  |  |  |
| Non-use | Reference |  | Reference |  | Reference |  | Reference |  |
| < 90 | 1.06 (0.97 – 1.15) | 0.200 | 1.11 (1.01 – 1.21) | 0.033 | 1.02 (0.91 – 1.14) | 0.033 | 1.13 (0.97 – 1.31) | 0.127 |
| 90 – 180 | 1.09 (0.97 – 1.23) | 0.155 | 1.07 (0.93 – 1.23) | 0.325 | 1.00 (0.84 – 1.21) | 0.325 | 0.89 (0.66 – 1.21) | 0.465 |
| 180 – 365 | 0.99 (0.89 – 1.09) | 0.836 | 0.95 (0.83 – 1.08) | 0.397 | 0.96 (0.80 – 1.14) | 0.621 | 0.98 (0.71 – 1.36) | 0.916 |
| 365 – 540 | 0.99 (0.86 – 1.15) | 0.956 | 0.85 (0.69 – 1.04) | 0.117 | 0.89 (0.63 – 1.27) | 0.529 | 1.02 (0.48 – 2.14) | 0.966 |
| ≥ 540 | 0.75 (0.61 – 0.91) | 0.005 | 0.64 (0.45 – 0.91) | 0.012 | 0.64 (0.15 – 1.08) | 0.072 | 0.88 (0.12 – 6.27) | 0.901 |
| P for trend | 0.261 |  | 0.092 |  | 0.302 |  | 0.846 |  |

cDDD, cumulative defined daily dose; HR, hazard ratio; aHR, adjusted hazard ratio; CI, confidence interval.

aHR was adjusted for age, sex, body mass index, income, smoking status, alcohol status, hypertension, diabetes, and baseline cholesterol level

**Supplementary Figure 1**. Illustration of the time-dependent survival analysis


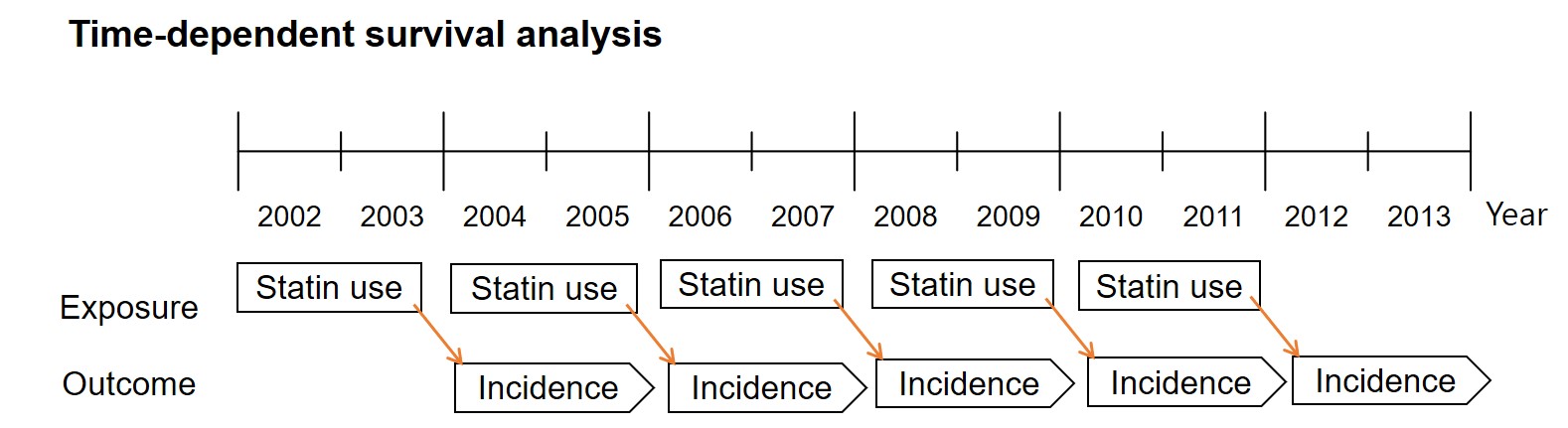

Supplement: Supplementary file 1 — Supplementary Information. [file 41598_2021_94803_MOESM1_ESM.docx]
